# A Divergent Platelet Transcriptome in Patients with Lipedema and Lymphedema

**DOI:** 10.3390/genes15060737

**Published:** 2024-06-04

**Authors:** Alliefair Scalise, Anu Aggarwal, Naseer Sangwan, Annelise Hamer, Suman Guntupalli, Huijun Edelyn Park, Jose O. Aleman, Scott J. Cameron

**Affiliations:** 1 Heart Vascular and Thoracic Institute, Department of Cardiovascular Medicine, Section of Vascular Medicine, Cleveland Clinic Foundation, Cleveland, OH 44195, USA; 2 Lerner Research Institute, Department of Cardiovascular and Metabolic Sciences, Cleveland Clinic Foundation, Cleveland, OH 44195, USA; 3 Holman Division of Endocrinology, New York University, New York, NY 10012, USA; jose.aleman@nyulangone.org; 4 Lerner College of Medicine, Case Western Reserve University, Cleveland, OH 44195, USA; 5 Department of Hematology, Taussig Cancer Center, Cleveland, OH 44195, USA

**Keywords:** lipedema, lymphedema, obesity, platelet, transcriptome, thrombosis

## Abstract

Lipedema and lymphedema are physically similar yet distinct diseases that are commonly misdiagnosed. We previously reported that lipedema and lymphedema are associated with increased risk for venous thromboembolism (VTE). The underlying etiology of the prothrombotic profile observed in lipedema and lymphedema is unclear, but may be related to alterations in platelets. Our objective was to analyze the platelet transcriptome to identify biological pathways that may provide insight into platelet activation and thrombosis. The platelet transcriptome was evaluated in patients with lymphedema and lipedema, then compared to control subjects with obesity. Patients with lipedema were found to have a divergent transcriptome from patients with lymphedema. The platelet transcriptome and impacted biological pathways in lipedema were surprisingly similar to weight-matched comparators, yet different when compared to overweight individuals with a lower body mass index (BMI). Differences in the platelet transcriptome for patients with lipedema and lymphedema were found in biological pathways required for protein synthesis and degradation, as well as metabolism. Key differences in the platelet transcriptome for patients with lipedema compared to BMI-matched subjects involved metabolism and glycosaminoglycan processing. These inherent differences in the platelet transcriptome warrant further investigation, and may contribute to the increased risk of thrombosis in patients with lipedema and lymphedema.

## 1. Introduction

Lower extremity swelling or edema can be attributed to multiple underlying etiologies, including venous hypertension, lipedema, and lymphedema. These conditions frequently co-exist and are easily mistaken for each other [1]. Two diseases affecting the limbs that are frequently misdiagnosed are lipedema and lymphedema.

Lipedema is a poorly understood and under-studied disease that almost exclusively impacts women. By physical exam, symmetric abnormal fat accumulation is present on the buttocks, thighs, and limbs, but excludes the feet [2]. Additionally, patients who have asymmetric fat distribution with lipedema present with pain and increased bruising [1]. Lymphedema, conversely, results from inflammation of the lymphatic vasculature causing abnormalities in circulating lymphatic fluid and manifesting as limb swelling. Unlike lipedema, lymphedema manifests with clear skin changes in the foot [3]. Primary lymphedema refers to intrinsic damage to the lymphatic system as a whole, whereas secondary lymphedema commonly develops following damage to a specific section of the lymphatic circulation or to individual lymph nodes [3].

Patients with lipedema and lymphedema often have comorbid obesity. Patients with obesity are known to be at risk for thrombosis (blood clotting) that may be precipitated by changes in both the transcriptome and translatome, and the consequent function of circulating platelets, as previously reported [4,5,6]. It was recently reported in patients with elevated BMI that lipedema and lymphedema are independent risk factors for venous thromboembolism (VTE) [7]. This suggests that elevated BMI alone may not be the driver of thrombosis [8]. It was also reported that the platelets-specific blood biomarker soluble glycoprotein VI (sGPVI) is elevated in the blood of patients with obesity as a function of increased BMI [9].

Given our understanding that the terminal pathway for thrombosis is the activation of circulating platelets and our previous demonstration that the transcriptome and translatome of the circulating platelet is highly adaptive in patients with certain diseases [10,11,12,13], we hypothesized that the circulating platelet phenotype, therefore, may differ in patients with elevated BMI, lipedema, and lymphedema from individuals with lower BMI.

As we previously observed in vascular diseases, the anucleate platelet still adapts using machinery available following budding from megakaryocytes, and the transcriptome offers biological insight into disease mechanisms that may lead to new therapeutics [10]. The purpose of this investigation was to compare the platelet transcriptome for changes in expression of individual genes and changes in groups of genes for corresponding biological pathways in patients with elevated BMI, in patients with lipedema, and in patients with lymphedema.

## 2. Materials and Methods

### 2.1. Study Approval

This study complies with the Declaration of Helsinki and was approved by the Cleveland Clinic Foundation RSRB for the analysis of blood and clinical variables associated with obesity, lipedema, and lymphedema. Each patient had previously received a clinical diagnosis of either lipedema or lymphedema by a clinician not involved in the study. Study participants were compared to age and BMI-matched healthy control volunteers. Pregnant patients were excluded from this study.

### 2.2. Diagnosis of Patients

The Herbst modified diagnostic criteria was used for the diagnosis of lipedema by an acknowledged expert in the care of patients with lipedema and lymphedema who was not involved in this study, [14,15]. Physical examinations to detect lipedema included the evaluation of subcutaneous fat distribution with lower-extremity ankle or upper-extremity elbow cut-off signs, palpation of subcutaneous fat, evaluation of Stemmer sign and dorsal foot hump, upper and lower extremity pulse examination and skin assessment of varicose veins, reticular veins, and corona phlebectatica. Furthermore, hypermobility testing using the Beighton score was performed. In appropriate situations, subsequent imaging studies including lower extremity venous duplex ultrasound, and lymphoscintigraphy, are sometimes performed in our institution to exclude the possibility of concomitant lymphatic insufficiency. A diagnosis of lymphedema is made by physical examination based on common and easily identifiable physical exam findings including a positive Stemmer sign, deep toe creases, edema, and skin thickening. This practice is supported by international experts that include the International Society of Lymphedema [16], the American Venous Forum, American Vein and Lymphatic Society and the Society for Vascular Medicine [17]. We occasionally employ lymphatic imaging modalities including ICG lymphography and lymphatic scintigraphy to support the diagnosis in select cases that require surgical correction. Each of our clinicians that establishes a diagnosis of lymphedema is board-certified through the American Board of Vascular Medicine and practices in a designated lymphedema center of excellence, as endorsed by the Lymphatic Education & Research Network.

### 2.3. Platelet Isolation and Transcriptomic Profiling

Platelets were isolated using standard techniques we describe in detail elsewhere [18]. After the second wash with Tyrode’s buffer, the possibility of genomic DNA contamination of platelets from residual leukocytes was removed by performing a depletion step with a standard kit containing an anti-human CD45 antibody (EasySep Human CD45 Depletion kit, StemCell Technologies, Cambridge, MA, USA). The final leukocyte-deplete washed platelet isolate was lysed in TRIZOL, allowing total RNA extraction that was then treated with DNAse using a standard kit (Qiagen RNEasy RNA extraction kit, Germantown, Md, USA). The final RNA yield following extraction was acceptable in each group: lipedema (17.1 ± 17.9 μg/μL), lymphedema (6.1 ± 3.3 μg/μL), and obesity (8.9 ± 2.7 μg/μL). Platelet messenger RNA sequencing (RNA-Seq) was performed using standard libraries available commercially at GENEWIZ (Waltham, MA, USA).

### 2.4. Bioinformatics and Statistical Analysis

Normalized count matrices were used for differential gene abundance using the DeSeq2 package (version 3.19). Pair-wise Log2FoldChange values were used for pathway enrichment analysis using the GO and KEGG database in clusterProfiler (version 3.04) and richR package (https://github.com/hurlab/RichR/) (accessed on 17 January 2023), respectively. The Gene ID listed in the annotation file Log2FoldChange for one group with respect to another was evaluated, and the adjusted *p*-value (padj; Benjamini–Hochberg adjusted *p*-value) was used prior to biological pathway analysis. Differentially expressed genes were used to identify the functional enrichment using GO enrichment ClusterProfiler package (version 3.04). The pre-processed RNA-seq data is available as an online supplement in Tables S1–S4.

## 3. Results

For the purposes of this study, we define obesity if the group BMI is >30 kg/m^2^. Heightened risk of thrombosis is established for patients with obesity, lipedema, or lymphedema [6,7,19]. Platelets are common terminal mediators of thrombosis, and adipose-tissue-reducing bariatric surgery changes the platelet transcriptome [20]. Therefore, we sought to determine whether the platelet transcriptome changes as a consequence of BMI, or if the platelet transcriptome and corresponding phenotype is specific to lipedema or lymphedema, given that BMI can influence platelet phenotype and function [4,6], and patients with lipedema generally, and also in this study, have obesity (mean BMI 37.7 ± 5.7 kg/m^2^; range: 27.9–46.5 kg/m^2^). Most patients enrolled with lymphedema also have obesity (mean BMI 34.1 ± 3.8 kg/m^2^; range: 26.1–41.0 kg/m^2^). Our control comparators recruited generally had elevated BMI (mean BMI 34.3 ± 5.7 kg/m^2^; range 24.2–43.7 kg/m^2^). To account for BMI class as a factor influencing the platelet transcriptome, our control group was further divided for separate analysis—one group who were overweight with Class I obesity (30.9 ± 5.3 kg/m^2^) and the second group with Class II obesity (37.7 ± 4.9 kg/m^2^). Age is also a reported factor that may change platelet phenotype and function irrespective of disease [21], so we carefully matched all patients in each age group within 5 years (Table 1). Physical exam features and the etiology of patients enrolled with lipedema and lymphedema were also recorded where available (Figure S1).

**Figure 1:**
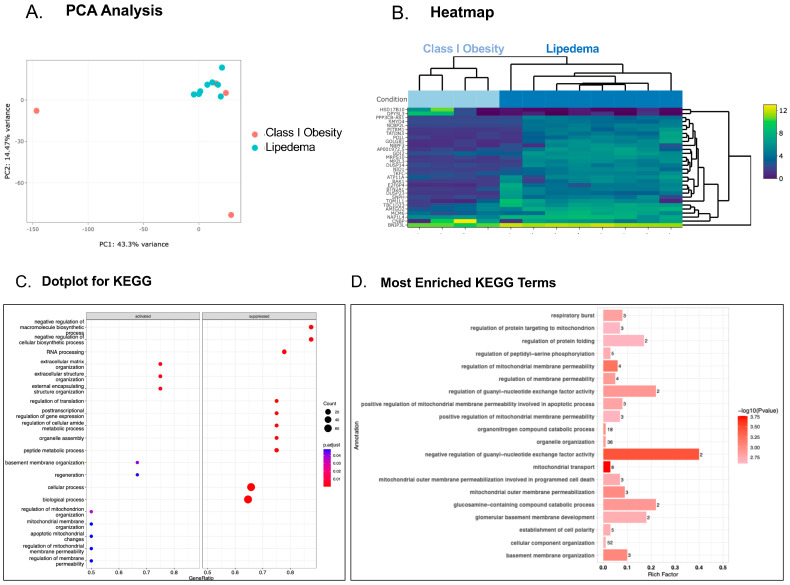
Evaluation of the platelet transcriptome in individuals with Class I obesity compared to individuals with lipedema. Principal component analysis (PCA) shows the separation between Class I obesity and lipedema along PC1 and PC2 (*p* < 0.01). The Bray–Curtis dissimilarity matrix was used to compute the inter-sample similarities. Statistical significance was estimated using PERMANOVA (**A**) and heatmap (**B**) of the top 30 differentially expressed genes in platelets from patients with Class I obesity (BMI 31 mg/kg^2^) compared with patients with lipedema (BMI 38 mg/kg^2^). KEGG dotplot (**C**) and GO (**D**) pathway enrichment analyses are listed and the most enriched pathways are illustrated.

**Figure 2:**
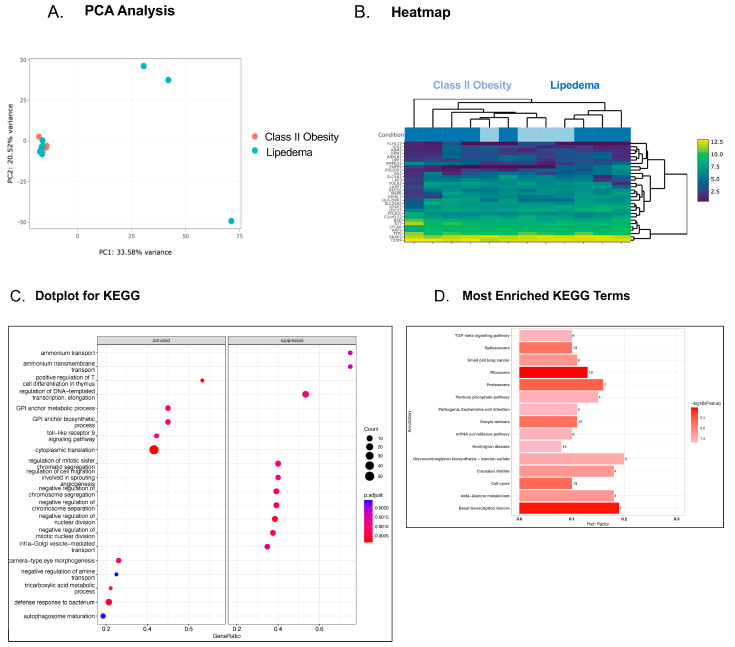
Evaluation of the platelet transcriptome in individuals with Class II obesity compared to patients with lipedema. Principal component analysis (PCA) shows the separation between Class II obesity and lipedema along PC1 and PC2 (*p* < 0.01). Bray–Curtis dissimilarity matrix was used to compute the inter-sample similarities. Statistical significance was estimated using PERMANOVA (**A**) and heatmap (**B**) of the top 30 differentially expressed genes in platelets from patients with Class II obesity (BMI 38 mg/kg^2^) compared with patients with lipedema (BMI 38 mg/kg^2^). KEGG dotplot (**C**) and GO (**D**) pathway enrichment analyses are listed, and the most enriched pathways are illustrated.

Platelet transcriptomic profiling in patients with Class I obesity compared to patients with lipedema revealed more genes that were upregulated (125 genes) than downregulated (11 genes) (Figure 1A,B). Cellular processes more active in platelets from patients with lipedema include those involved in the rearrangement of the extracellular matrix, the cytoskeleton, and the activity of small G-proteins via guanine nucleotide exchange factor (GEF) (Figure 1C,D). All of these biological processes influence platelet reactivity, and in particular, GEFs are known to activate and degranulate platelets [22]. In contrast, the interrogation of the platelet transcriptome in patients with Class II obesity compared to patients with lipedema with very similar BMI revealed fewer differences, and none that reached statistical significance, though perhaps more genes clustering around biosynthetic and metabolic processes and increased glycosaminoglycan (GAG) synthesis were observed in patients with Class II obesity compared with lipedema (Figure 2A–D).

**Figure 3:**
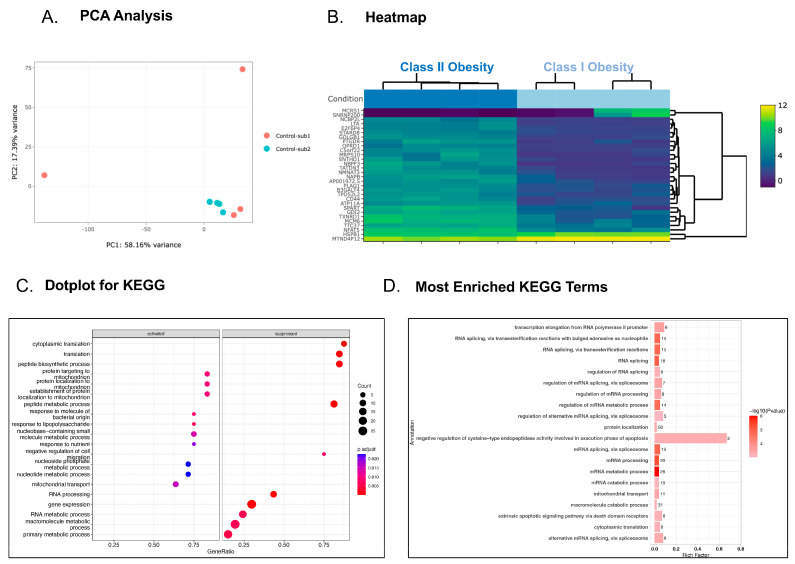
Evaluation of the platelet transcriptome in patients with Class I compared to Class II obesity. Principal component analysis (PCA) shows the separation between Class I obesity and Class II obesity PC1 and PC2 (*p* < 0.01). Bray–Curtis dissimilarity matrix was used to compute the inter-sample similarities. Statistical significance was estimated using PERMANOVA (**A**) and heatmap (**B**) of the top 30 genes in platelets from patients with obesity Class I (BMI 31 mg/kg^2^) compared with patients with obesity Class II (BMI 38 mg/kg^2^). KEGG dotplot (**C**) and GO (**D**) pathway enrichment analyses are listed, and the most enriched pathways are illustrated.

Class I obesity control subjects had an average BMI of 31 kg/m^2^ in comparison to Class II obesity control subjects who had an average BMI of 38 kg/m^2^. Platelet RNA sequencing was reported to show changes in patients following bariatric surgery, suggesting that elevated BMI from adipocytes reprograms platelets that are derived from bone marrow megakaryocytes [20]. Comparing the transcriptomes of patients with Class I and Class II obesity in this study, there was a striking difference in the platelet transcriptome, with many upregulated genes (225 genes) and a few downregulated genes (73 genes) in platelets from patients with increased BMI (Figure 3A,B). There was a strong bias toward gene clustering in platelets for processes that promote apoptosis and negatively regulate transcription and translation in patients with Class II obesity (Figure 3C,D).

**Figure 4:**
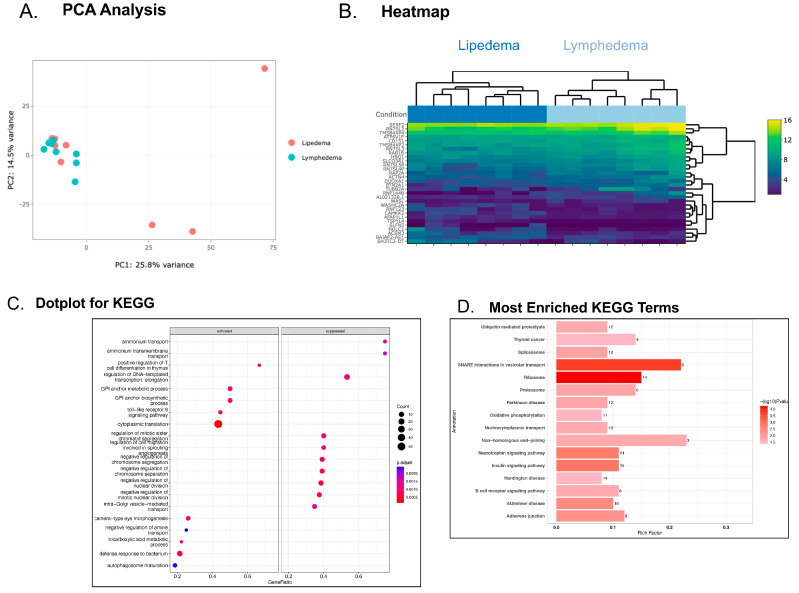
Evaluation of the platelet transcriptome in individuals with lipedema compared to individuals with lymphedema. Principal component analysis (PCA) shows the separation between lipedema and lymphedema along PC1 and PC2 (*p* < 0.01). Bray–Curtis dissimilarity matrix was used to compute the inter-sample similarities. Statistical significance was estimated using PERMANOVA (**A**) and heatmap (**B**) of the top 30 differentially expressed genes in platelets from patients with lymphedema (BMI 34 mg/kg^2^) compared with patients with lipedema (BMI 38 mg/kg^2^). KEGG dotplot (**C**) and GO (**D**) pathway enrichment analyses are listed and the most enriched pathways are illustrated.

Comparing the transcriptome of platelets in patients with lipedema revealed more subtle differences in gene expression compared with platelets isolated from patients with lymphedema (Figure 4A,B), which showed a clearer program favoring the post-translational modification of proteins by glycolipids, protein dephosphorylation, cytoplasmic protein translation, and regulated exocytosis of platelet granules in lymphedema. Conversely, in platelets from patients with lymphedema, there were decreased angiogenesis signals and decreased signals for inhibiting mitosis compared with lipedema. While this was a very small sample of patients with lipedema and lymphedema, we did perform a limited retrospective analysis for clinically-significant thrombosis. We determined for lymphedema that three patients (28%) had a history of DVT, and one patient (12.5%) had a history of stroke. We determined for lipedema that one patient (12.5%) had a history of PE, one patient (12.5%) had a history of stroke, and one patient (12.5%) had a history of MI.

## 4. Discussion

We discovered that platelets isolated from patients with lipedema and lymphedema have a divergent transcriptome that is mostly driven by obesity. Elevated BMI is a comorbidity often found in patients with lipedema and lymphedema and observed in 94% of our patients in this study. While patients with lipedema and lymphedema have a similar incidence of thrombosis [7], platelets from patients with lipedema demonstrated a divergent transcriptome when compared to platelets isolated from patients with lymphedema. Observed differences involved genes and biological pathways responsible for protein synthesis, glycosaminoglycan synthesis, granule exocytosis, and protein dephosphorylation.

Because circulating platelets are myeloid-derived entities that bud from precursor megakaryocytes, they are pre-programmed to the host in which they circulate, and offer important general biological insights into diseases. Although platelets are anucleate, they have a dynamic and active transcriptome and translatome that allows them to adapt to their unique host milieu. A previous study demonstrated that plasma extracellular vesicles have an increased quantity of platelet factor 4 (PF4) in patients with lipedema and disorders of the lymphatic system [23]. PF4 is expressed in the top 1% of genes in platelets and could be a useful platelet-derived biomarker for identifying patients with lipedema and disorders of the lymphatic system.

The evaluation of the transcriptome in the lipedema platelet revealed biological pathways involving regulation of protein targeting to the mitochondria by promoting mitochondrial membrane permeability and apoptotic processes when compared to Class I obesity platelets. Two specific genes that were found to be involved include *HSD17B10* and *BNIP3L*. Platelet transcriptomics revealed that the gene encoding 17-β-Hydroxysteroid dehydrogenase (*17B-HSD*) that is found on the X-chromosome (HSD10) is downregulated in patients with lipedema when compared to patients with Class I obesity. 17β -HSD is a mitochondrial enzyme involved in the oxidation of fatty acids and branched-chain amino acids, and may be involved in the metabolic derangement of adipose tissue [24,25]. 17β -HSD contributes to the oxidation of fatty acids and branched-chain amino acids, and possesses the ability to utilize ketone bodies as substrates [25]. Patients with lipedema are known to have abnormal metabolism and a decreased response to calorie-restrictive diets, unless forced into ketosis by a ketogenic diet [26]. The resistance to a typical low-calorie weight loss dietary intervention that patients with lipedema experience may be attributed to the downregulation of mitochondrial 17B-HSD, making it more difficult for patients with lipedema to respond to a calorie deficit. *BNIP3L*, a gene found to be significantly upregulated in patients with lipedema, is heavily involved in regulating the mitophagic process by encoding a proapoptotic outer membrane protein on mitochondria that aids in recruiting and enhancing the formation of autophagosomes [27]. *BNIP3L* activity has been altered in various diseases including cardiovascular diseases, metabolic disorders, neurologic diseases, and cancer [27]. However, in lipedema, the upregulation of *BNIP3L* suggests a critical role in regulating mitophagy by encoding an important proapoptotic mitochondrial membrane protein, which promotes the formation of autophagosomes. Mitophagy is the pathway by which mitochondria are cleared from the cell in response to signaling cues from environmental and intracellular stressors [27]. As platelet activation is an energy-intensive process, mitochondrial dysfunction and, as a result, cellular bioenergetics are intimately tied to aberrant platelet reactivity [28,29,30]. A study performed by Rihman and colleagues found that impaired mitochondrial gene expression is linked to platelet activation [30]. Because of this result, the upregulation of *BNIP3L* may explain the increased rates of VTE experienced by these patients.

Platelet transcriptomics revealed that the transforming growth factor β (TGF-β) signaling pathway, the pentose phosphate pathway, and the synthesis of the glycosaminoglycan keratan sulfate varied significantly in lipedema platelets in comparison to Class II obesity platelets. TGF-β is a multifunctional cytokine that is abundant in platelets and released in platelet granules upon activation, and it influences cell proliferation, migration, and differentiation, as well as extracellular matrix production [31]. This molecule serves as an important modulator in wound healing due to its influences on fibrosis. TGF-β is also known to be positively correlated with rates of thrombosis by promoting hypercoagulable states [32]. TGF-β accomplishes this by impairing fibrinolysis and aggravating endothelial dysfunction [32]. The upregulation of TGF-β in lipedema platelets provides new insight into the increased VTE risk in patients with lipedema.

Another pathway that was divergent in lipedema platelets from Class II obesity controls was the pentose phosphate pathway (PPP). The PPP is an anabolic pathway that occurs in parallel with glycolysis and is critical in maintaining carbon homeostasis, generating precursors for nucleotide and amino acid biosynthesis, and producing NADPH, which plays important roles in redox homeostasis [6]. In platelets, the PPP is stimulated during aerobic glycolysis, resulting in an increased production of NADPH, which is subsequently used as a substrate for NADPH oxidase [33]. This reaction releases reactive oxygen species (ROS) into the platelet and triggers a signaling cascade to induce the activation of platelet surface integrins, ultimately leading to platelet aggregation and thrombosis [29,33].

The extracellular environment of patients with lipedema appears to be unique, with tissue injury resulting in poor wound healing [34]. One report suggests glycosaminoglycans, or GAGs, are increased in the extracellular matrix of patients with lipedema. This may contribute to the edema and abnormal lymphatic propulsion and drainage reported in lipedema [35,36]. It is interesting that, in our analysis, lipedema platelets showed a significantly reduced signal for GAGs compared to Class II obesity platelets, suggesting differences in which platelets may contribute to tissue remodeling in lipedema outside those observed in patients with similar BMI due to obesity. Since platelet factor 4 (PF4) is known to bind to GAGs and promote extracellular matrix remodeling [37,38], mechanisms of platelet α granule secretion may contribute to tissue remodeling in patients with lipedema, and antiplatelet therapy may limit these pathological process. Additionally, glycosaminoglycans are responsible for binding sodium and water in the extracellular fluid [36]. In doing so, glycosaminoglycans reduce the edematous effect produced by an increase in extracellular fluid. KS has the highest binding specificity of the glycosaminoglycan molecules at a rate of 67%, and the upregulation of this molecule offers an explanation for the lack of edema experienced by patients with lipedema, despite a known increase in extracellular fluid in accordance with increasing BMI [35,36]. This finding suggests that KS is a key player in extracellular matrix organization and developmental biology as a whole.

When comparing the platelet transcriptome in patients with lipedema with that of patients with lymphedema, we determined the genes were surprisingly more similar than different. If one considers that the platelet functions as a circulating biosensor, as we previously reported [10,11,12,39], we would anticipate similar alterations in transcriptional pathways in related vascular disorders. However, we did discover some unique biological pathways altered in platelets from patients with lipedema compared with lymphedema. For example, we discovered alterations in soluble N-ethylamide-sensitive-factor (NSF) attachment protein receptor (SNARE) interactions and vesicular transport in lipedema platelets when compared to lymphedema platelets. NSF has been shown by several groups to control platelet degranulation and thrombosis in platelets [40,41,42]. SNARE molecules also regulate the fusion of vesicles with their target membranes, including the careful regulation of the platelet secretome, ultimately impacting platelet aggregation and thrombosis [43]. SNARE proteins are present on both the vesicle membranes (v-SNARE) and target membranes (t-SNARE) so as to control membrane fusion and facilitate the exocytosis of platelet granules [42]. SNARE-mediated exocytosis from platelets releases α-granules, dense granules, and lysosomal granules into the circulation [40]. The molecules contained in these granules promote platelet recruitment, activation, adherence, and aggregation [40]. NSF is a partial player in the mediation of exocytosis of platelet granules; more specifically, NSF regulates the release of α-granules from platelets. When α-granule release is inhibited in vivo, decreased adhesion is observed. Nitric oxide is a known inhibitor of thrombosis, and it accomplishes this by moderating NSF assembly [41]. SNARE-coded proteins were found to be upregulated in patients with lipedema when compared to patients with lymphedema. This finding indicates that nitric oxide production is decreased in patients with lipedema, resulting in increased NSF assembly, increased α-granule release, and ultimately increased rates of VTE in these patients.

Obesity is an established risk factor for platelet activation, which can be reduced by a decrease in adiposity [19]. Additionally, obesity is known to be associated with elevated platelet counts in women [44], which do not improve with rapid weight loss [45]. Of the 30 genes identified by platelet transcriptomics, 27 were found to be significantly upregulated in type II obesity in comparison to type I obesity. These genes included, but are not limited to, *STARD8*, *GOLGB1*, and *NAPB*. *STARD8* regulate RhoA, a GTPase required for platelet granule secretion [46]. A variant of *GOLGB1* was found to decrease platelet activation, indicating that an upregulation of *GOLGB1* in Class II obesity controls is associated with increased thrombosis [47]. *NAPB* is a gene that encodes NSF-attachment protein β [48]. As previously stated, NSF regulates the exocytosis of platelet granules and enhances thrombosis. Because of these results, it is reasonable to identify a positive correlation between weight gain and platelet reactivity. Comparing patients with Class I obesity to those with Class II obesity, biological pathways regulating programmed cell death (apoptosis) were significantly different. Healthy platelets and platelets examined from patients with thrombotic diseases were previously reported to show changes in apoptosis [49,50]. The evaluation of the platelet transcriptome revealed that platelets from Class I obesity controls exhibited higher levels of apoptosis. Obesity is known to be associated with elevated platelet counts in women [44].

In summary, we describe here, for the first time, a distinct platelet transcriptome in patients with lipedema and lymphedema compared to subjects with obesity. While obesity is an established risk factor promoting thrombosis in a variety of conditions, and patients with lipedema and lymphedema often have an elevated BMI, we have shown in prior work that these patients have residual thrombotic risk even when accounting for elevated BMI [7]. The platelet is a critical mediator of primary hemostasis, but when reprogrammed in thrombotic conditions, it may promote pathologic thrombosis. Herein, we outline multiple biological pathways in the platelets of patients with lipedema and lymphedema compared to patients with obesity that may mediate the observed residual thrombotic risk. It is concerning that, for such a small sample of patients, we observed that several had a history of DVT, PE, stroke, and MI, suggesting a tendency toward a thrombotic phenotype. Further work characterizing the precise mechanisms that promote thrombotic risk in lipedema and lymphedema are warranted, and will allow clinicians to better care for these patients.

Limitations of this study should be considered when interpreting the findings and planning future investigation in this area. Firstly, cross-sectional designs only provide a snapshot of data at a single point in time, making causality conclusions or disease progression difficult. To understand the evolution of platelet transcriptome in lipedema, lymphedema, and obesity-related conditions, longitudinal studies that evaluate an adaptive translatome would be informative. This is especially important given that previous studies have proven that the changes in gene expression are not always reflected by changes in the same platelet protein due to the de-coupling of the processes that control the transcriptome and the proteome [51,52]. Second, the study’s sample size and demographic diversity may limit the generalizability of the observations. Additional confounding factors including medication use, co-morbid medical conditions, and lifestyle choices were not fully controlled.

## 5. Conclusions

We previously highlighted in a large clinical cohort that both lipedema and lymphedema confer an increased risk of thrombotic events, including VTE. As platelets are the most common terminal mediators of in vivo thrombotic responses, and are often aberrantly programed in thrombotic conditions, we aimed to evaluate the transcriptome in lipedema and lymphedema platelets. The distinct transcriptomic profiles of platelets in lipedema and lymphedema compared to BMI- and age-matched control cohorts may mechanistically underlie the observed heightened risk of thrombotic complications in this population of patients. Further investigations into the precise mechanisms of VTE in patients with lipedema and lymphedema are warranted, and these patients should be closely monitored and appropriately treated for thrombotic complications.

## Figures and Tables

**Table 1:** Basic demographics of patients studied.

	Lipedema (*n* = 8)	Lymphedema (*n* = 8)	Obesity I (*n* = 4)	Obesity II (*n* = 4)
Age, y; mean (SD)	57.1 (9.7)	50 (21)	53.3 (5.3)	51.3 (14.9)
Female gender, n (%)	8 (100)	8 (100) ^a^	4 (100) ^b^	4 (100) ^c^
BMI, kg/m^2^; mean (SD)	37.9 (6.2)	34.1 ^d^ (5)	30.9 ^e^ (5.3)	37.7 ^f^ (4.9)

*p*-values for BMI vs. lipedema: ^a^ = 0.42, ^b^ = 0.48, ^c^ = 0.44. *p*-values for age vs. lipedema: ^d^ = 0.20, ^e^ = 0.08, ^f^ = 0.93.

## Data Availability

The original contributions presented in the study are included in the article/Supplementary Material, further inquiries can be directed to the corresponding author.

## Supplementary Materials

The following supporting information can be downloaded at: https://www.mdpi.com/article/10.3390/genes15060737/s1, Figure S1: Physical exam features of patients enrolled with lipedema and lymphedema. A. Etiology and exam features of lipedema where known. B. Etiology and exam features of lymphedema where known; Table S1: Lipedema vs. Class I Obesity; Table S2: Lipedema vs. Class II Obesity; Table S3: Class I vs. Class II Obesity; Table S4: Lipedema vs. Lymphedema.

## Abbreviations

Body mass index (BMI), venous thromboembolism (VTE), deep vein thrombosis (DVT), pulmonary embolism (PE).
