# Supplementary material for: A Divergent Platelet Transcriptome in Patients with Lipedema and Lymphedema
Source: Genes (Basel). 2024 Jun 4;15(6):737. doi: 10.3390/genes15060737 (PMC11202821; doi:10.3390/genes15060737)
Supplement: Supplementary file 1 [file genes-15-00737-s001.zip › genes-3007091-Suppl. Figure S1.pdf]

A. **Lipedema**

Lipedema Patient #	Etiology	Stage	Pitting Edema in Legs (grade)
**1**	Unknown	2	2+ edema
**2**	Unknown	Unknown	trace
**3**	Unknown	2	trace
**4**	Unknown	Unknown	Nonpitting
**5**	Pregnancy	Unknown	Nonpitting
**6**	Unknown	2	Nonpitting
**7**	Familial	3	Nonpitting
**8**	Menarche	Unknown	Nonpitting

B. **Lymphedema**

Lymphedema Patient #	Etiology	Primary/Secondary	Stage	Pitting Edema in Legs (grade)
**1**	Elevated BMI	Secondary (obesity)	2	Left leg only, non-pitting
**2**	Lymph node removal	Secondary (ovarian mass removal)	1b	2+ edema
**3**	Venous insufficiency	Secondary (venous reflux)	Unknown	2+ edema
**4**	Venous insufficiency	Secondary (venous reflux)	Unknown	1+ edema
**5**	Unknown	Primary	2	None
**6**	Unknown	Primary	Unknown	1+ edema
**7**	Post-partum	Secondary (pregnancy)	Unknown	1+ edema
**8**	Unknown	Primary	2	2+ edema

Figure S1. Physical exam features of patients enrolled with lipedema and lymphedema. **A**. Etiology and exam features of lipedema, where known. **B.** Etiology and exam features of lymphedema, where known
